# The fluid management and hemodynamic characteristics of PiCCO employed on young children with severe hand, foot, and mouth disease—a retrospective study

**DOI:** 10.1186/s12879-021-05889-z

**Published:** 2021-02-25

**Authors:** Fengyun Wang, Xinhua Qiang, Suhua Jiang, Jingsong Shao, Bin Fang, Lixin Zhou

**Affiliations:** 1 Department of Critical Care Medicine, The First People’s Hospital of Foshan, Lingnan Avenue North 81, Shiwan, Chancheng, Foshan, 528000 China; 2 Department of Pediatric Intensive Care Units, The First People’s Hospital of Foshan, Foshan, China

**Keywords:** Hand, foot, and mouth disease, Enterovirus 71, Pulse indicator continuous cardiac output, Acute pulmonary edema, Fluid management

## Abstract

**Background:**

Hand, foot, and mouth disease (HFMD) is an acute infectious disease caused by human enterovirus 71 (EV71), coxsackievirus, or echovirus, which is particularly common in preschool children. Severe HFMD is prone to cause pulmonary edema before progressing to respiratory and circulatory failure; thus hemodynamic monitoring and fluid management are important to the treatment process.

**Methods:**

We did a review of young patients who had been successfully treated in our department for severe HFMD, which had been caused by EV71. A total of 20 patients met the inclusion criteria. Eight cases were monitored by the pulse indicator continuous cardiac output (PiCCO) technique, and fluid management was administered according to its parameters. With regard to the treatment with PiCCO monitoring, patients were divided into two groups: the PiCCO group (8 patients) and the control group (12 patients). The groups were then compared comprehensively to evaluate whether PiCCO monitoring could improve patients’ clinical outcomes.

**Results:**

After analysis, the findings informed that although PiCCO failed to shorten the length of ICU stay, reduce the days of vasoactive drug usage, or lower the number of cases which required mechanical ventilation, PiCCO did reduce the incidence of fluid overload (*p* = 0.085) and shorten the days of mechanical ventilation (*p* = 0.028). After effective treatment, PiCCO monitoring indicated that the cardiac index (CI) increased gradually(*p* < 0.0001), in contrast to their pulse (P, *p* < 0.0001), the extra vascular lung water index (EVLWI, *p <* 0.0001), the global end diastolic volume index (GEDVI, *p* = 0.0043), and the systemic vascular resistance index (SVRI, *p <* 0.0001), all of which decreased gradually.

**Conclusion:**

Our study discovered that PiCCO hemodynamic monitoring in young children with severe HFMD has some potential benefits, such as reducing fluid overload and the duration of mechanical ventilation. However, whether it can ameliorate the severity of the disease, reduce mortality, or prevent multiple organ dysfunction remain to be further investigated.

## Introduction

HFMD is an acute infectious disease caused by human enterovirus 71 (EV71), coxsackieviruses (CAV), echovirus 18, or some other viruses, which is predominant in preschool children [1, 2]. EV71 ranks high in the etiology of HFMD [3], and is easily exacerbated by encephalitis. When the brain stem is affected, HFMD may cause neurogenic pulmonary edema, which is common, but fatal if not promptly treated. Additionally, the incidence of CAV A6 and A10 infection has been increasing in recent years [4–6]. Research shows that severe HFMD is among the top 10 causes of death in pediatric patients in China [7, 8]. Chinese pediatric experts consensus divided HFMD into five stages, according to which stage 3 and stage 4 are severe, thus often need intensive care [9] because the deterioration of stage 4 children often accelerated rapidly, with some of them dying suddenly as a result of central nervous system and/or pulmonary-circulatory failure [10–12]. In stage 3 and stage 4, fluid resuscitation may be required if the patient’s heart rate is fast, serum lactate is high, or blood pressure is dropping. Nevertheless, in severe HFMD, the occurrence of lung edema is common [13]. Pulse indicator continuous cardiac output (PiCCO) monitoring is a widely used invasive method to monitor hemodynamics in critical patients. Here, we reviewed pediatric cases, involving EV71-induced severe HFMD, at stages 3 and 4, which cases were successfully treated in our department. With regard to treatment with PiCCO monitoring, patients were divided into two groups: the PiCCO group and the control group. We presented the hemodynamic characteristics of these patients, and compared the two groups comprehensively to evaluate whether PiCCO monitoring could improve their clinical outcomes.

## Methods

Participants: the diagnosis and staging of severe HFMD caused by EV71 were based on the consensus of Chinese pediatricians [9]. Their diagnosis of severe EV71 HFMD, complicated with respiratory and circulatory failure, occurred at stages 3 and 4. At stage 3, the pediatricians found that patients’ heart and respiration, blood pressure, and the coldness and dampness in the extremities all increased. In stage 4, patients showed cyanotic, pink phlegm; bloody sputum; hypotension; altered states of consciousness, or oliguria; and, many eventually advanced to respiratory and circulatory failure.

From October 2011 to September 2015, the cases of 20 children, successfully treated for stages 3 and 4 HFMD, were reviewed. For this study, treatment success was defined as successful discharge without relapse within 90 days. All the methods were accomplished in accordance with official guidelines and regulations. This study was approved by the Ethics Review Committee, the First People’s Hospital of Foshan and conducted in accordance with the provisions of the Declaration of Helsinki (Approval No. 2011FS105). Informed consent to participate was obtained from the parents/guardians of all patients.

Inclusion criteria: 1) Clinical manifestations that met the diagnostic criteria of stages 3 and 4 HFMD [10]. 2) Positive EV71 nucleic acid test. 3) Implantation of a PiCCO catheter within 6 h of admission to the ICU. Exclusion criteria: 1) Premies. 2) Children with congenital cardiopulmonary disease. 3) Death within 90 days of admission.

In view of treatment with PiCCO monitoring, patients were divided into two groups: the PiCCO group (8 patients) and the control group (12patients). Antiviral, corticosteroids, diuretics, cardiotonic agents and other supportive treatments were administered for patients in both groups whenever necessary. In the PiCCO group, the hemodynamic parameters were mainly monitored by PiCCO. In control group, the noninvasive blood pressure, respiratory rate, pulse, electrocardiogram and peripheral oxygen saturation were monitored by ECG monitor. Fluid input and urine output were monitored every 8 h and arterial blood gas was tested once daily in both groups. Relevant laboratory tests such as myocardial enzyme spectrum, atrial natriuretic peptide (BNP) and renal function were tested as necessary.

The PiCCO monitoring procedure: patients’ femoral artery was implanted with an PiCCO catheter under the guidance of bedside ultrasound. The PiCCO catheter was manufactured by Pulse Medical System SE, Germany; model, pv2014l16n; outer diameter, 4F; total length, 16 cm. As part of the process, their pulse and invasive blood pressure were continuously monitored by pressure sensor; additionally, every 6 h, 20 ml of 4 °C saline was injected into the conduit to monitor hemodynamic parameters such as cardiac output index (CI); stroke volume index (SI); extra vascular lung water index (EVLWI); global end diastolic volume index (GEDVI); and, systemic vascular resistance index (SVRI), and other such indicators. This saline injection was repeated thrice, and the average value of the parameters was obtained.

Outcome measures: 1) Age; sex; weight; and severity. 2) Baseline body temperature; blood pressure; heart rate; systolic blood pressure; CI; ejection fraction (EF); systolic blood pressure; cases of vasoactive drugs usage; cases of mechanical ventilation; fluid overload; and acute kidney injury (AKI) cases. 3) Baseline pH value; oxygenation index; blood lactate; serum creatinine (Cre); serum TnI; serum CK-MB; days of vasoactive drugs usage; length of mechanical ventilation days or length of ICU stay. 4) In the PiCCO group, at 0 h, 24 h, 48 h and 96 h, the pulses; CI; SI; SVRI; EVLWI; and, GEDVI were all dynamically monitored.

Statistical analyses: Graphpad 8 was used to analyze all the data. Continuous variables with abnormal distributions were expressed as medians and interquartile (IQR). T-test, one-way anova, and chi-square test were applied accordingly. *P* < 0.05 was considered statistically significant.

## Results

### Baseline characteristics of young children with EV-A71 induced severe HFMD

There was no significant difference in age, weight, gender and severity between the PiCCO group and the control group. No significant difference was found between the two groups about the baseline heart rate, EF, CI (ultrasonic results), and systolic blood pressure. The detailed data was presented in Table 1.

**Table 1:** Baseline characteristics of young children with EV-A71 induced Severe HFMD

Characteristics	PiCCO(*n* = 8)	Control (*n* = 12)	p
Age (month)	18 [12.25, 28.5]	20.3 [13.5, 25.4]	0.875
Male	5	6	0.582
Severity			
Stage 3, n	3	7	0.361
Stage 4, n	5	5	
Weight	9.7 [7.3,10.8]	10.5 [8.7, 12.2]	0.405
Temperature	39.5 [39.2,39.8]	39.4 [39.0,39.9]	0.562
Heart rate (bpm)	187 [178,196]	185 [181,194]	0.747
LVEF	39 [36.3,42.5]	40.5 [37.5,42.8]	0.668
CI	3.27 [2.86,3.51]	3.34 [2.95,3.48]	0.913
Systolic blood pressure	107 [83,116]	111 [91,125]	0.551

Abbreviations: *PiCCO* Pulse indicator continuous cardiac output, *LVEF* Left Ventricular Ejection Fraction, *CI* Cardiac Index

### Laboratory indexes and clinical outcomes

Arterial blood gas showed that young children with severe HFMD had metabolic acidosis; there was no difference between the PiCCO group and control group. The oxygenation index of both groups was low at the time of admission, indicating potential respiratory failure. Patients’ blood lactate was high on admission, indicating that there were microcirculatory disorders and tissue metabolic dysfunction. The oxygenation index and blood lactate didn’t differ between the two groups. Serum Cre, TnI, and CK-MB were not significantly different for the two groups, suggesting that there was no difference in baseline kidney or heart status. The incidence of AKI, the cases of vasoactive drugs usage, and cases of mechanical ventilation had no significant difference between the two groups. However, the cases of fluid overload tended to be less in the PiCCO group, and the result was close to statistical significance. Compared with the control group, the duration of mechanical ventilation in the PiCCO group was shortened, but there was no significant difference in the length of ICU stay and days of use of vasoactive drugs. Data elaborating on this is presented in Table 2.

**Table 2:** Laboratory indexes and clinical outcomes of young children with EV-A71 induced Severe HFMD

Outcomes	Picco(*n* = 8)	Control (*n* = 12)	p
pH	7.18 [7.05, 7.37]	7.21 [7.07, 7.31]	0.744
PaO2/FiO2, mmHg	187 [152.5, 217.8]	195 [161.5, 210]	0.534
LAC, mmol/L	3.23 [2.56, 4.74]	3.43 [2.63,4.56]	0.671
Cre, μmol/L	55.0 [28.1, 79.8]	58 [28.5, 75.5]	0.791
TNI, μg/L	0.15 [0.05, 0.38]	0.13 [0.06, 0.40]	0.797
CK-MB, IU/L	28 [18, 54]	25 [16, 59]	0.749
***Cases of vasoactive agents***			
Sodium nitroprusside	2	3	0.999
Urapidil hydrochloride	5	8	0.848
Dopamine	2	1	0.306
Norepinephrine	3	4	0.848
Days of vasoactive drugs usage	7.5 [6.0, 10.0]	7.8 [6.3,9.5]	0.714
Cases of AKI	3	5	0.852
Cases of MV	4	5	0.714
Cases of fluid overload	1	6	0.085
Length of ICU stay, days	11 (9, 16)	11.3 (9.3, 15.8)	0.847
Duration of MV, days	6.9 [4.7,8.8]	9.3 [5.9, 12.7]	0.028

Abbreviations: *PiCCO* Pulse indicator continuous cardiac output, *LAC* Lactate, *Cre* Creatinine, *TNI* Troponin-I, *CK-MB* creatine kinase-MB, *AKI* Acute kidney injury, *MV* mechanical ventilation

### Hemodynamic characteristics monitored by PiCCO in severe HFMD caused by EV71

**Fig. 1:**
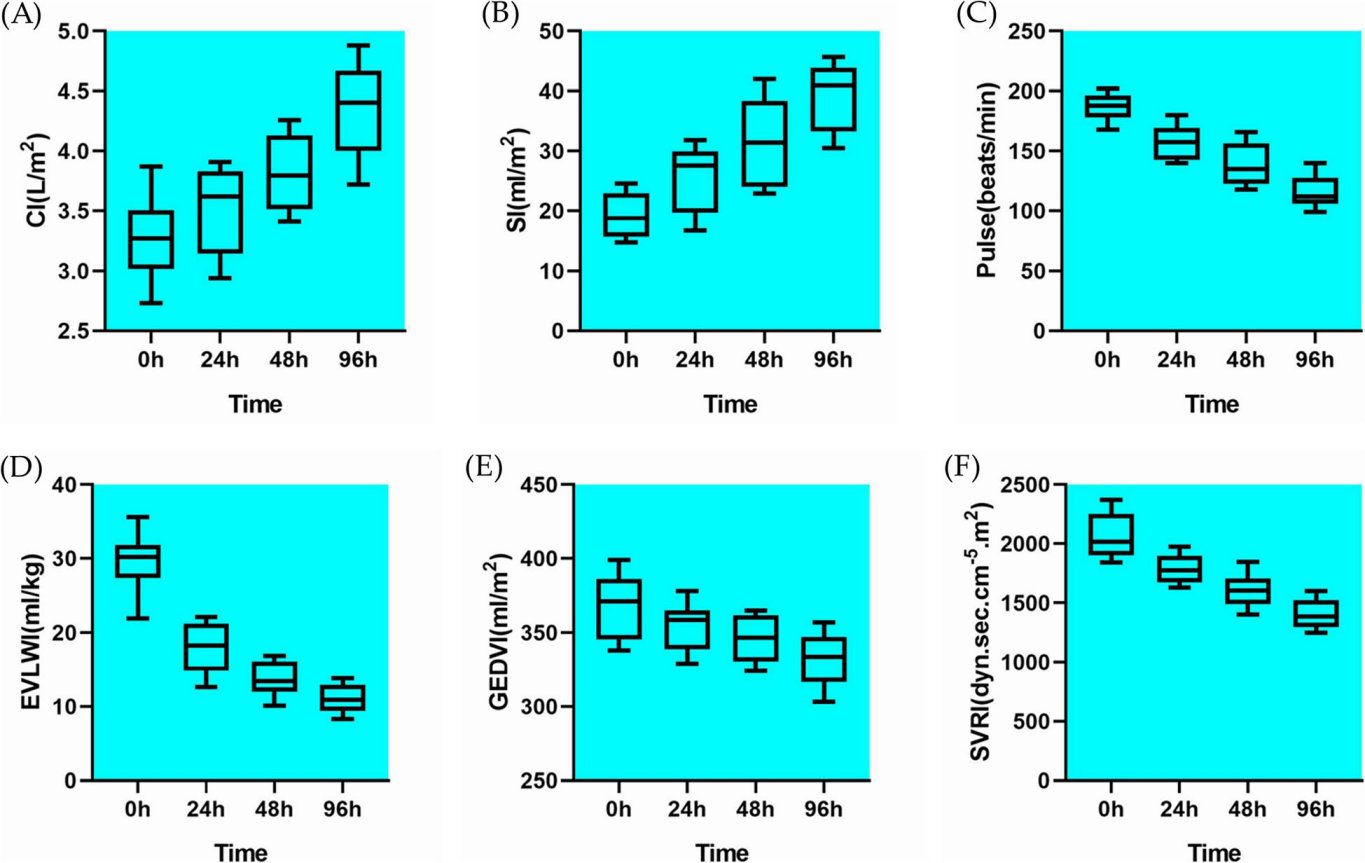
The dynamic monitoring of (**a**) CI, (**b**) SI, (**c**) Pulse, (**d**) EVLWI, (**e**) GEDVI and (**f**) SVRI by PiCCO. CI(*p* < 0.0001) and SI (*p <* 0.0001) increased gradually, whereas pulse(*p <* 0.0001), EVLWI(*p <* 0.0001), GEDVI(*p* = 0.0043) and SVRI(*p <* 0.0001) decreased through time. Abbreviations: cardiac output index CI; stroke volume index SI; extra vascular lung water index EVLWI; global end diastolic volume index GEDVI; and, systemic vascular resistance index SVRI

**Fig. 2:**
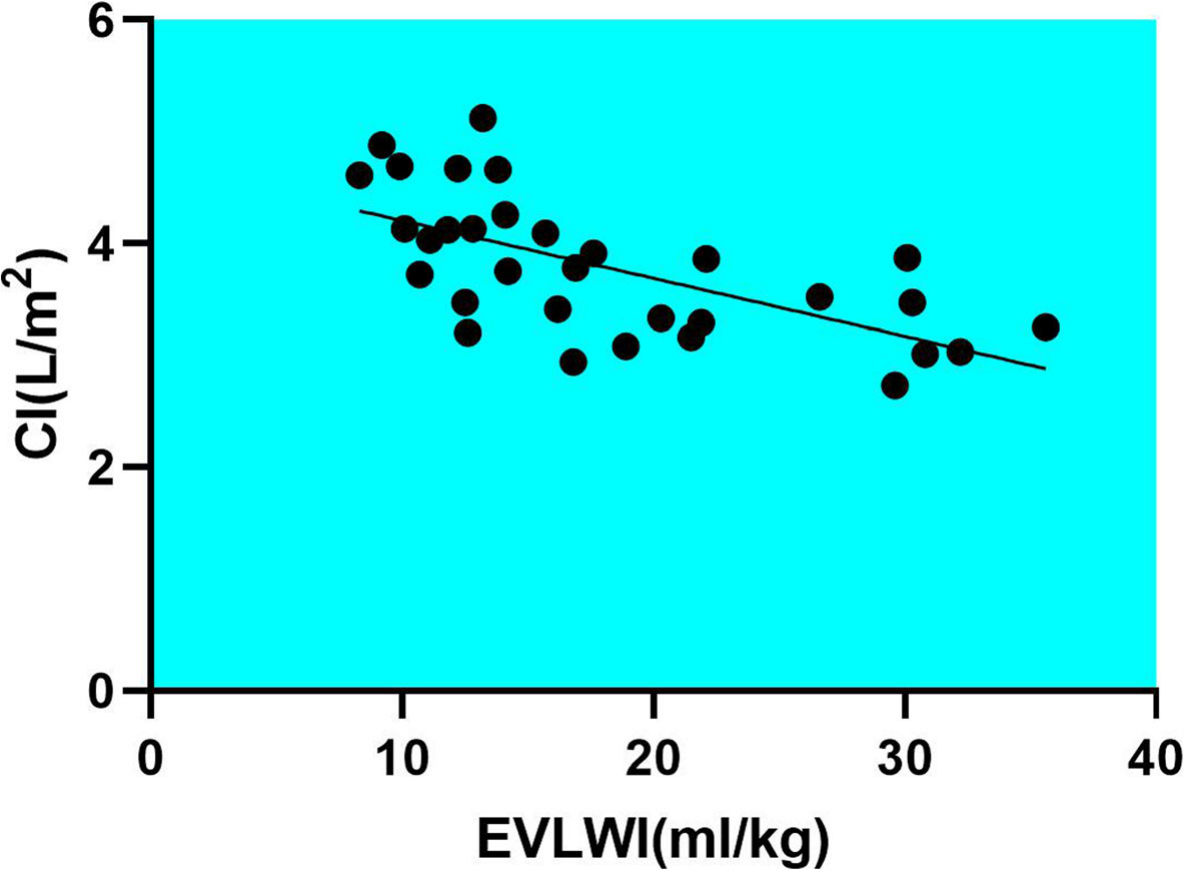
The linear regression analysis of CI and EVLWI, EVLWI was correlated with CI. Equation: Y = − 0.05171*X + 4.723, *p <* 0.0001. Abbreviations: cardiac output index CI; extra vascular lung water index EVLWI

PiCCO monitoring showed that due to timely and effective treatment, the patients’ pulse gradually slowed down and tachycardia was relieved. After the treatment with antiviral, cardiotonic, and diuretics, the patients’ CI and SI increased, and EVLWI decreased. Meanwhile, GEDVI decreased gradually, which indicated that the heart was potentially dilated on admission. With the passage of time and the improvement of cardiac function, the SVRI decreased gradually. Consistently, the patients’ limbs increased in warmth and peripheral perfusion improved. The data is exhibited in Fig. 1. Through linear regression, EVLWI was found to be correlated with CI. With the gradual recovery of CI, EVLWI gradually decreased (Fig. 2).

## Discussion

The purpose of PiCCO monitoring in this study was to investigate whether it can be useful for better fluid management and facilitate rapid relief of the disease. Detecting the risk factors of acute pulmonary edema, in young children with HFMD as early as possible, and administering effective and timely treatment have an important role in reducing mortality. To our knowledge, this is the first report of PiCCO hemodynamic monitoring in preschool children.

HFMD**—**characterized by fever and a rash of hands, feet, mouth and buttocks, is a condition that is highly infectious and predominant in preschoolers. With timely diagnosis and treatment, the patients can recover fully [14]. However, a few patients have experienced central nervous system dysfunction [15] and acute pulmonary edema, which are the main causes of death [16, 17]. According to the HFMD guideline, severe cases at stages 3 and 4 should be admitted to ICU promptly. EV71-induced HFMD, in particular, is inclined to be worsened by encephalitis. When the brain stem is involved, HFMD is likely to give rise to neurogenic pulmonary edema [18–21]. Some of the stage 3 and 4 HFMD patients needed vasoactive agents to sustain normal blood pressure and were considered hemodynamically unstable, thus, PiCCO technique was considered.

The patients included in our study were divided into a PiCCO group and a control group, to assess the value of applying PiCCO. Though PiCCO did not shorten the length of ICU stay, or reduce the cases of mechanical ventilation or the cases of vasoactive drugs usage, some meaningful results were still obtained. 1) PiCCO monitoring reduced the cases of patients with fluid overload, which is probably a result of the real-time monitoring of EVLWI, GEDVI, CVP and other volume-related indicators. 2) PiCCO monitoring helped decrease the duration of mechanical ventilation, which may stem from the fact that PiCCO enabled the optimization of fluid management and the rapid relief of pulmonary edema. Dynamic monitoring suggested that EVLWI decreased gradually over time, which confirmed the reduction of pulmonary edema. 3) Through linear regression analysis, it was found that with the gradual recovery of CI, EVLWI gradually decreased, which may indicate that viral myocarditis cannot be excluded in severe HFMD, and there is the possibility that cardiogenic pulmonary edema existed in the course of the disease.

PiCCO is a routine invasive technique in the critical care field, but it is seldom applied in general wards. Consequently, PiCCO catheter are usually inserted to patients after their transfer to ICU. However, we agree that if PiCCO could be implanted earlier, the fluid management could be done better, which may be beneficial to stage 3 and 4 severe HFMD patients. In our department, 4 °C saline was injected every 6 h to measure hemodynamic parameters of PiCCO. It’s a habitual routine, which is not mandatory. However, as PiCCO is only applied to potentially circulation unstable patients, longer intervals are not considered unless the condition of the patients has been improved significantly.

In 2001, Wu et.al reported that in the 5 children with EV71-caused HFMD, Swan Ganz catheter monitoring and magnetic resonance showed that severe HFMD was complicated with brainstem encephalitis and acute pulmonary edema [22]. The results indicated that tachycardia and low SI were the two most common clinical manifestations. However, due to the normal pulmonary artery wedge pressure (PAWP) and CI, there was no significant increase in vessel resistance. Therefore, Wu et al. concluded that the cause of acute pulmonary edema was not cardiogenic. PiCCO hemodynamics monitoring has been widely used in clinical settings, especially in ICU [23, 24]. Assessment of extravascular lung water (EVLW) by pulmonary thermal-dilution technology [25] has been confirmed by many basic experiments and its utilization makes bedside EVLW evaluation a routine requirement in critical care medicine [24, 26].

In addition to treatment such as dehydration, anti-viral and anti-bacterial infections, and mechanical ventilation, some patients in this study were also administered with immunoglobulin, and/or methylprednisolone. With reference to PiCCO monitoring, the children also received cardiotonic, and/or diuretics therapy. Fluid management by PiCCO succeeded as patients’ pulse slowed, CI and SI increased, and EVLWI, GEDVI and SVRI decreased. In this study, the corresponding decrease of lung fluid may be interpreted as the recovery of heart function. The decline of GEDVI after treatment may suggest that when the patients reached stages 3 or 4 diagnosis, the volume of the left ventricle became enlarged [27]. Hence, based on the findings of this study, it is possible to speculate that the cause of pulmonary edema in children with severe HFMD is not only neurogenic but also cardiogenic.

Similar to the study of Wu et.al [22], the 8 patients in PiCCO group showed tachycardia and low cardiac output on admission, and CI was slightly lower than the normal range. The results of PiCCO showed that CI decreased, while GEDVI increased, which was consistent with the results of some studies—indicating that the left ventricle dilated and its systolic function was impaired in children with severe HFMD. At present, HFMD is still common in southern China [28, 29], and severe HFMD is still the main cause of death in children [30]. This study confirmed the feasibility of PiCCO hemodynamic monitoring in young children. Although there is no known reference interval of parameters of PiCCO for young children, the trend of PiCCO parameters still can provide markers for guiding fluid management. Volume management, under the guidance of PiCCO, may reduce the fluid overload in children with severe HFMD, relieve pulmonary edema more swiftly, and lower the duration of mechanical ventilation.

Finally, the limitations of this study are as follows: 1) This was a retrospective study, with a small number of cases included, thus the conclusions need to be verified by larger studies. 2) Only successfully treated cases were included, hence the impact of PiCCO on mortality has not been evaluated. 3) PiCCO catheter implant is an invasive procedure; with the rise of non-invasive hemodynamic monitoring techniques, especially in children, its prospects are not so optimistic. 4) Due to limited medical resources, synchronous re-examination of cardiac ultrasound was not accomplished for both groups; therefore, some parameters of PICCO and ultrasound results may be quite different. 5) With the popularity of vaccines and hygiene improvement, the incidence of HFMD is decreasing in Guangdong, and follow-up studies may be difficult to carry out.

## Conclusion

In severe HFMD, PiCCO hemodynamic monitoring is feasible. Although there is no known reference interval for young children at present, the trend of dynamic monitoring is still of significant value for clinical diagnosis and fluid management. This retrospective found that PiCCO monitoring can reduce the fluid overload and duration of mechanical ventilation. However, the improvement of the severity of the disease, the impact on mortality, and the prevention and treatment of MODS by PiCCO monitoring need to be further verified.

## Data Availability

The datasets used and/or analyzed in this study are available from the corresponding author on reasonable request.

## Abbreviations

HFMD: Hand, foot, and mouth disease
EV71: Enterovirus 71
PiCCO: Pulse indicator continuous cardiac output
CI: Cardiac output index
SI: Stroke volume index
EVLWI: Extra vascular lung water index
GEDVI: Global end diastolic volume index
SVRI: Systemic vascular resistance index
EF: Ejection fraction
